# Practical Hints

**Published:** 1848-10

**Authors:** 


Practical Hints.—Under this head we design, in each number of the Journal, to say a few words on miscellaneous subjects connected with our profession. We shall aim to say something new, and avoid, so far as it is practicable, a repetition of old facts and principles, already recorded and familiar to our readers.

We shall not presume always to draw from our own store-house exclusively; but whenever we do allude to hints and valuable suggestions gathered from without the pale of our sanctum, ample credit will be given for the same.

If we should at times fail in our efforts to say something new, our apology must be, that the facts we set forth have never been recorded, or at least, have never come under our observation. If, however, we should dilate upon truths and principles already familiar to some of our readers, the circumstance must be attributed to that *coincidence of discovery* which may be regarded as one of the leading features of the spirit of our age, as is manifested in the discovery of great facts and principles, simultaneously, at different parts of the world. We find it abundantly illustrated in our own profession, in the difficulty we often experience in ascribing a discovery to its true source; a difficulty which we can only reconcile, by the reflection, that it as justly belongs to several, as that it does to any one individual.

All of us, more or less, have in the store-house of our experience and research, many pet discoveries and inventions, which we have ever regarded as our own exclusive creations, and which are linked indisputably to our own individuality. Many of them we have cherished for years. Some of them have risen, step by step, through successive elaborations, until, in our fancy, they have arrived to the fullest perfection; while others were but in their embryo, and waited the development which we have as yet been unable to give them; and we have revelled in the confidence of the security of our bantlings, while, at the same time, we have looked forward with complaisant satisfaction, to the time when the world would acknowledge to us their obligations for the same. And yet, how often, on visiting the sanctum of an ingenious friend, or the laboratory of one of the masters of our art, have we found our pet discoveries provokingly anticipated, with all of the advantages which the perfecting experience of *years* could give them! Some of our cherished inventions, which we had but half matured, we find here glorying in a completeness of perfection, which we would have considered as presumptive to anticipate.

So much for the illustration of the principle. But genius does not always flow in the same channel; and though coincidences like the above may occasionally occur, yet with all of us, there are many things which will bear the severest test of scrutiny, and yet will come back to us, endorsed with a world-sanctioned authority *as our own!*

It may be objected, that at times we go too minutely into detail, or descend into the A, B, C, of the art. To this we reply, that it has perhaps been a fault of our Journal, with its professed character, that its articles often have been too general to be practical; and that often, where our readers have looked for detail and clear elucidation of our science, they have only found theory and speculation.

A generous detail is often as valuable to its recipient, as the knowledge of a great general principle, which indeed might be valueless without it.

But while we have an eye to this, we shall endeavor never to tread on over familiar ground, nor let verboseness take the place of practical sentiment.

*Impressions in Plaster.*—The usual method of taking impressions in plaster is doubtless generally known to most of our readers; but a few further, and perhaps not altogether familiar hints on the subject, may not be unacceptable to those who fail of complete success in this department

The article of gypsum or plaster of paris, differs essentially in its character; first, according to the locality from which it is obtained; and secondly, in the manner in which it is prepared for use.

Pure gypsum is found to consist of lime, 28; sulphuric acid, 40; water, 18; its full equivalent being a fraction over 86. This is the article most commonly in use in our country under the name of plaster of paris; though our market is mostly supplied from Nova Scotia. Pure gypsum is found, however, in various parts of Europe. The Nova Scotia plaster is obtained by grinding the crystals of gypsum (selenite) to a powder in a mill, and then heating it in a kettle, up to a little above 300° Fahr., (it will boil before it reaches this temperature,) when its two equivalents of water of crystallization are expelled, and it is fit for use. When mixed with water to the consistence of cream, it absorbs its two equivalents of water, and becomes recrystallized, though in an opaque form. If heated to a temperature over 400° Fahr., the particles become indurated, and it no longer possesses the property of absorbing water. But this article is comparatively soft and brittle, is easily decomposed by heating, and is incapable of withstanding the influences of the weather.

The true plaster of paris, or calcareous gypsum, is of course imported from the vicinity of Paris. It contains about 12 per cent. of carbonate of lime. It is prepared by being *baked* and afterwards ground. When in the form of paste, it is exceedingly plastic, and when consolidated, remarkably hard and tough, owing no doubt to the presence of the carbonic acid contained in the carbonate of lime. It is used in Paris extensively, as an out-of-door building material, where it is exposed with impunity to all of the influences of the weather. It will also withstand a high temperature of heat without being impaired. All of these peculiarities render it infinitely superior to the Nova Scotia plaster for our purpose of taking impressions.

The calcareous gypsum, or Paris plaster, is readily distinguished from the Nova Scotia, in that the latter is of a very clear white color, inclining to blue; it has also a feeling of roughness or grittiness, while the Paris plaster is of a decided yellowish white, and has a soapy feeling when passed through the fingers. Its plasticness when mixed with water, will at once distinguish it from any other kind. We assure our readers that after making a trial of this article for the purpose mentioned, they would have no inclination to use any other. The plaster should always be finely pulverized and sifted, and the mouth wiped dry, previous to taking impressions.

In taking impressions in plaster, it often happens that the mould obtained is entirely perfect, except those parts corresponding with the highest point of the roof of the mouth; this imperfection is occasioned by air being concentrated in that locality, in consequence of the cup-shape formation of the jaw, given to it by the alveolar ridge. This can easily be avoided, and success always rendered certain. Previous to putting the plaster-holder into the mouth, place a small wedge-shaped piece of plaster in a semi-plastic state, in the highest part of the roof of the mouth, its point of course touching first; as you press it up, it will gradually fill at every point around from the centre, without the possibility of there being any air-hole or imperfection thus far. This will generally fill the roof of the mouth down nearly on a level with the bottom of the alveolar ridge. Your holder, charged with comparatively a small quantity of plaster, is now introduced into the mouth; as it is gradually pressed upwards, it readily unites with the plaster already there, and flowing around the anterior surface of the gums, the union is complete at every quarter. On removing the plaster, you are always gratified to find the impression perfect in every respect.

But it oftentimes, if not generally, occurs, especially if the above directions are followed in full, that great difficulty is experienced in removing the impression from the mouth, in consequence of atmospheric pressure. Sometimes we have been obliged to resort to the severest measures to separate the one from the other. No lifting one side or the other, no pressing backwards or forwards would suffice. And when after a good display of

"bone and sinew" we have finally brought it away, tinctured with many a sanguineous stain, we have found a half-way apology for our severity in the comforting exclamation, that it was a "*remarkably* good impression." But this is no late thing with us; we date it back to the *dark ages* of our practice; to our credit be it spoken, we soon discovered a remedy for this, which has ever since proven as infallible as it is satisfactory.

In all of our holders for upper sets, we have a small hole punctured in them, about one line in diameter, at a point corresponding to the centre of the palatine arch. Just before filling one of these with plaster, we close up this orifice with a thin piece of sheet wax. Immediately on the plaster being pressed to its place in the mouth, we take a small piece of wire, and with it penetrating the wax, we pass it upward through the plaster, until it reaches the roof of the mouth; it is thus suffered to remain until the plaster is nearly or quite hard, when it is withdrawn. By this means, an air passage is formed to the centre of the impression, where the vacuum is always most complete, and the holder may be removed without the least difficulty.

The crystallizing or hardening process of the plaster is wonderfully facilitated by using pure rain water, into which a small quantity of common salt or alum has been dissolved. While the use of hard water simply, retards its setting, dissolving gum arabic or glue, in this last, will protract its setting to a very remarkable extent.

We should avoid leaving our plaster in a damp place, or it may absorb moisture and soon become unfit for use; this can be dispelled however by heating it over again. Even plaster that has once been cast into forms, when pulverized and baked anew, is fit for use again.

The imperfections occasioned by air-holes, in casting the plaster into moulds for the purpose of obtaining models, may be easily obviated by first placing a small quantity of thin batter into them, (we generally use a finer sort for this,) and then *blowing* down upon it a small current of air from the mouth, until we have displaced it, so that the surface of the mould, immediately under this stream of air, is nearly or quite discernible. We trace this little circlet, made by the force of the air from the mouth, displacing as we go, all over the surface of the mould. By this means, you drive every hidden bubble from its retreat, and at the same time force the plaster into the most delicate depressions of the mould. You then fill up the casting to the desired height, and on separating the model from the mould, you cannot but be delighted with the delicate perfection of your work.

This method of blowing upon the plaster when in the form of a thin paste, is practiced by sculptors and our best artists in plaster; by which means they obtain perfect impressions of the most delicate and elaborate works of art.

The charming medallions and relievos in plaster, which occasionally come to our eyes, with their lines as sharp and delicate, and an exquisiteness of finish, as though they had come direct from the hand of the original artist, and were indeed archetypes themselves, were cast in this manner, for such perfection can be gained in no other way.—*Cazenovia Ed.*

